# Comparative metabolic profiling of *Vitis amurensis* and *Vitis vinifera* during cold acclimation

**DOI:** 10.1038/s41438-018-0083-5

**Published:** 2019-01-01

**Authors:** Fengmei Chai, Wenwen Liu, Yue Xiang, Xianbin Meng, Xiaoming Sun, Cheng Cheng, Guotian Liu, Lixin Duan, Haiping Xin, Shaohua Li

**Affiliations:** 1 Key Laboratory of Plant Germplasm Enhancement and Specialty Agriculture, Wuhan Botanical Garden, Chinese Academy of Sciences, Wuhan, P.R. China; 2 Beijing Key Laboratory of Grape Sciences and Enology, CAS Key Laboratory of Plant Resources, Institute of Botany, Chinese Academy of Sciences, Beijing, P.R. China; 3 University of Chinese Academy of Sciences, Beijing, P.R. China; 4 Key Laboratory of Plant Molecular Physiology, Institute of Botany, Chinese Academy of Sciences, Beijing, P.R. China; 5 State Key Laboratory of Crop Stress Biology in Arid Areas, College of Horticulture, Northwest A&F University, Yangling, Shaanxi P.R. China; 6 International Institute for Translational Chinese Medicine, Guangzhou University of Chinese Medicine, Guangzhou, P.R. China

**Keywords:** Secondary metabolism, Abiotic

## Abstract

*Vitis amurensis* is a wild *Vitis* plant that can withstand extreme cold temperatures. However, the accumulation of metabolites during cold acclimation (CA) in *V*. *amurensis* remains largely unknown. In this study, plantlets of *V. amurensis* and *V. vinifera* cv. Muscat of Hamburg were treated at 4 °C for 24 and 72 h, and changes of metabolites in leaves were detected by gas chromatography coupled with time-of-flight mass spectrometry. Most of the identified metabolites, including carbohydrates, amino acids, and organic acids, accumulated in the two types of grape after CA. Galactinol, raffinose, fructose, mannose, glycine, and ascorbate were continuously induced by cold in *V. amurensis*, but not in Muscat of Hamburg. Twelve metabolites, including isoleucine, valine, proline, 2-oxoglutarate, and putrescine, increased in *V. amurensis* during CA. More galactinol, ascorbate, 2-oxoglutarate, and putrescine, accumulated in *V. amurensis*, but not in Muscat of Hamburg, during CA, which may be responsible for the excellent cold tolerance in *V. amurensis*. The expression levels of the genes encoding β-amylase (BAMY), galactinol synthase (GolS), and raffinose synthase (RafS) were evaluated by quantitative reverse transcription-PCR. The expression *BAMY* (*VIT_02s0012 g00170*) and *RafS* (*VIT_05s0077 g00840*) were primarily responsible for the accumulation of maltose and raffinose, respectively. The accumulation of galactinol was attributed to different members of GolS in the two grapes. In conclusion, these results show the inherent differences in metabolites between *V. amurensis* and *V. vinifera* under CA.

## Grapes: staying out in the cold

Studying how the wild grape *Vitis amurensis* copes with cold may help in breeding grape varieties that can grow at lower temperatures. To prevent frost damage, plants can increase cells’ sugar and protein contents, lowering the freezing point, and preventing ice crystal formation. *V. amurensis*, a wild grape native to Asia, grows well at low temperatures, but its cold-tolerance compounds have yet to be identified. Haiping Xin and Shaohua Li at the Chinese Academy of Sciences and co-workers profiled the compounds present in *V. amurensis* and *V. vinifera* cv. Muscat of Hamburg, a wine grape, following cold stress. *V. amurensis* cells were richer in a dozen compounds, including amino acids, organic acids, and putrescine, a component of the odor of decaying flesh. These results may help in developing grape varieties with increased cold tolerance.

## Background

Low temperature stress is a major determinant of plant growth and development. This condition not only affects the geographical locations of crops but also causes significant losses in their productivity^1^. Investigations on the regulatory mechanisms underlying low temperature stress responses in plants are essential for crop breeding. Plants show increased freezing tolerance following exposure to low non-freezing temperatures, a phenomenon known as cold acclimation (CA)^2,3^. CA in plant cells leads to a series of physiological and biochemical changes, including increased scavenging of reactive oxygen species^4^, increased membrane stability^5^, and accumulated soluble sugars^6,7^ and proline^6,8^. These responses ultimately increase freezing tolerance^9^.

Mass spectrometry (MS)-based metabolomic techniques identify comprehensive metabolites during the cold condition in plants^10,11^. Metabolomics, together with transcriptomics and proteomics, provides a major tool for the characterization of postgenomic processes^12^. Metabolomics involves detecting and quantifying metabolic changes with techniques such as gas chromatography–MS (GC–MS)^13,14^, liquid chromatography–MS (LC–MS)^15,16^, and nuclear magnetic resonance (NMR) spectroscopy^17^. GC–MS-based metabolite profiling offers a good balance of sensitivity and reliability and is considerably more sensitive than NMR spectroscopy and more robust than LC–MS^18^. GC–MS is particularly effective in the analysis of primary metabolites, specifically those involved in central carbon metabolism. The metabolic profiles have been analyzed in many plants exposed to low temperature stress. These plants include the model plant *Arabidopsis*^19,20^ and other species such as *Oryza sativa*^21,22^, *Triticum aestivum*^23^, *Fragaria* sp.^24^, *Thellungiella salsuginea*^25^, and *Haberlea rhodopensis*^26^. Low temperature stress leads to numerous changes in the metabolic profile of plants. Specific metabolites accumulate in cold-acclimated plants that correlate with the cold tolerance of plants^27–29^. For example, this stress increases the levels of carbohydrates, such as sucrose, galactinol, and raffinose^30^. The levels of amino acids, such as proline, organic acids, and tricarboxylic acid (TCA) cycle intermediates also increase. The metabolites in cold-tolerant accessions, cultivars, or species differ from those in cold-sensitive ones under low temperature stress, and these metabolites possibly contribute to cold tolerance^20^. In general, stress-tolerant plants express more metabolites, such as proline and soluble sugars, than stress-sensitive plants^31^. However, stress-sensitive plants accumulate few stress-associated metabolites, which are not sufficient to resist against cold damages. For example, analysis of the metabolic profile of *Arabidopsis* accessions with different levels of freezing tolerance revealed that compatible solutes, including proline and raffinose, contribute to freezing tolerance^20^. Similarly, metabolic profiling of the cold-tolerant species *H. rhodopensis* and of *Thellungiella* and *Arabidopsis* indicated that *H. rhodopensis* is pre-adapted to cold by constitutive accumulation of high levels of protective metabolites, such as galactinol and raffinose^26^.

Grape is an important fruit economically worldwide^32^. Grape is primarily grown in temperate and subtropical regions, which have low temperatures that negatively affect the production and quality of grapes. Rapid temperature drop in late fall, freezing temperatures in mid-winter, and early spring frost seriously damage grapevines and cause fruit production losses^33,34^. Investigations on cold stress responses and regulatory pathways are important for breeding new grape cultivars with preeminent cold tolerance.

*Vitis amurensis* is an important wild grape germplasm resource that originated from eastern Asia. This *Vitis* species shows strong tolerance to extremely low temperatures, even less than −40 °C^34,35^, and has been widely used as a parent in grape breeding for selecting cold-tolerant cultivars^36^. To elucidate the mechanism underlying low temperature adaptation, transcriptome modifications under cold treatment in *V. amurensis* have been analyzed, and a subgroup of cold stress-related genes has been identified^33,36^. In *V. amurensis*, the functions of several cold-induced genes, such as *CBF*^37^, *ICE*^38^, *ERF*^39^, and *GRAS*^40^, have also been reported. Although these findings increase our knowledge of cold stress response in *V. amurensis*, the metabolite changes in grape plants under CA remain unclear. Discovering the CA-induced metabolome modifications under CA in grapes, particularly in *V. amurensis*, may not only help to understand the mechanisms underlying low temperature adaptation in grapes but also provide the essential data to explain the excellent cold tolerance in *V. amurensis*.

In the present study, plantlets of *V. amurensis* and *Vitis vinifera* cv. Muscat of Hamburg (with less cold tolerance when compared with *V. amurensis*) were treated at 4 °C for 24 and 72 h. GC coupled with time-of-flight MS (GC–TOF–MS) was used to identify the metabolite changes in the leaves of the two grape species. The expression levels of genes encoding for β-amylase (BAMY), galactinol synthase (GolS), and raffinose synthase (RafS) were evaluated by quantitative real-time PCR (qRT-PCR) analyses. The relationships between the gene expression and the accumulation of the metabolites were analyzed. The possible contribution of CA-related metabolites during cold tolerance in *V. amurensis* was also discussed.

## Materials and methods

### Plant materials

Micropropagated seedlings of *V. amurensis* (collected from Changbai Mountain in Jilin Province, China) and *V. vinifera* cv. Muscat of Hamburg were cultivated in a chamber at the Wuhan Botanical Garden of the Chinese Academy of Sciences. The seedlings were grown on 1/2 B5 medium with 30 g L^−1^ sucrose at 26 °C with a 16-h light/8-h dark photoperiod. Low temperature treatments were performed by placing 40-day-old plantlets in another growth chamber with the same parameters except for temperature, which was set at 4 °C. The leaves were collected after 0 (used as the control), 24, and 72 h and immediately frozen in liquid nitrogen. Then, the samples were ground into fine powder and stored at −80 °C. Six and three replicate samples were collected at specific time points for each species for metabolome and qRT-PCR analyses, respectively.

### Measurement of metabolites

Leaf extracts were prepared as previously described by Weckwerth et al.^41^. Ground samples (100 mg) were transferred into 2 mL centrifuge tubes. A total of 1.5 mL of buffer containing methanol/chloroform/water (5:2:2, v/v/v) and ribitol (5 mg mL^−1^, an internal standard) was added into the tubes. The samples were then incubated at 37 °C for 2 h. Subsequently, the tubes were centrifuged at 12,000 × *g* for 10 min, and the supernatant was decanted into a 2 mL screw-top glass tube. Then, the tubes were placed into a vacuum concentrator at 30 °C for 2 h to dry the extracts. The extracts were dissolved in 50 μL of methoxamine hydrochloride and then incubated at 37 °C for 2 h. Then, the samples were derivatized with 80 μL of *N*-methyl-*N*-trimethylsilyl trifluoroacetamide for 30 min at 37 °C.

The well-prepared samples were analyzed with a gas chromatograph (6890N; Agilent Technologies, Santa Clara, CA, USA) coupled with a Pegasus IV time-of-flight mass spectrometer (LECO Instruments, St. Joseph, MI) that used a VF-5 capillary column (Varian, USA). Helium was used as the carrier gas with a flow rate of 1 mL min^−1^. The column temperature was maintained at 60 °C for 1 min, increased to 310 °C at a rate of 8 °C min^−1^, and then held for 15 min. Injection and ion source temperatures were 250 °C and 200 °C, respectively. Energy was set at −70 eV in an electron impact mode. MS data were acquired in a full scan mode with an m/z range of 50–600 after a solvent delay of 6 min.

Peak identification and quantification were performed using Chroma TOF (version 4.34) of the LECO Corporation. In brief, the data processing method included ‘Baseline,’ ‘Peak Find,’ and ‘Calculate Area/Height’^41–43^. For these processes, the peak width was set at 6 s. A maximum of detected peaks over a signal/noise threshold of 20 was used. Compound identification was based on searching the mass spectra in the standard NIST08 library^41^. Unique fragment ions for each individual metabolite were used for area calculation and manually corrected when necessary^44^. The mass retention time paired with the highest relative abundance was chosen to represent each metabolite. The amount of metabolites was normalized to plant mg fresh weight (FW) and then by area of internal references.

### Annotation of members of the BAMY, GolS, and RafS gene families in grapes

Members of the BAMY gene family in grape were identified using Hidden Markov Model (HMM), BLASTP program and NCBI-Conserved Domain Data (CDD) search. In brief, the HMM (HMMER, http://www.hmmer.org) from Pfam was downloaded to identify related homologous genes^45^. Then, we searched its homologous genes in the *V. vinifera* genome (http://plants.ensembl.org/index.html) with the model using HMM. The threshold value was set to 1^e−15^. Seven members of the BAMY gene family in grape were identified by the NCBI-CDD (http://www.ncbi.nlm.nih.gov/Structure/cdd/wrpsb.cgi) search (Supplementary Figure S1)^46^. Members of GolS and RafS gene families were identified based on Sun et al. (Supplementary Table S4).^47^

### qRT-PCR analysis

Total RNA of grape leaves was extracted using an RNAprep Pure Plant Kit (DP432; Tiangen Biotech, Beijing, China). RNA quality and concentration were detected through agarose gel electrophoresis and spectrophotometry (NanoDrop 2000; Thermo Scientific, USA). The complementary DNA was synthesized using HiScript® II Reverse Transcriptase (Vazyme Biotech Co., Nanjing, China) and then subjected to qRT-PCR on an Opticon thermocycler (CFX Connect Real-Time System; Bio-Rad, Hercules, CA) using SYBR Green PCR master mix (Vazyme Biotech Co, Nanjing, China) in accordance with the manufacturer’s instructions. *VviActin* (accession: EC969944) was used as the reference gene, and specific primer pairs for relevant genes were designed using Primer-BLAST (http://www.ncbi.nlm.nih.gov/tools/primer-blast/, Supplementary Table S5). The specificity of designed primers was verified through gel electrophoresis. The PCR reactions were set under the following conditions: 95 °C for 10 min, 40 cycles of 95 °C for 10 s and 60 °C for 30 s. Each sample was analyzed with three biological and technical replicates. The relative expression levels of the target genes were examined using the 2−ΔΔCt method. Significant difference was determined by *t*-test (**P < 0.01, *P < 0.05, *n* = 3) using the R program.

### Statistical analyses

Detected samples of metabolites were replicated six times; means and calculated standard errors (S.E.) are reported (Supplementary Table S1). The ratio of the average normalized peak areas for the metabolite between CA and pre-stress > 1 with *P* < 0.05 increased in response to cold stress, and whereas that between CA and pre-stress < 1 with *P* < 0.05 decreased. *P*-values of metabolite data were calculated using a two-tailed Student’s *t*-test comparing inter- and intra-genotype differences to cold stress at 0, 24, and 72 h. *T*-test was performed using R (version 3.1.1), and principal component analysis (PCA) was conducted using SIMCA-P (version 13.0; Umetrics, Umea, Sweden).

## Results

### PCA of metabolites under cold treatment

**Fig. 1:**
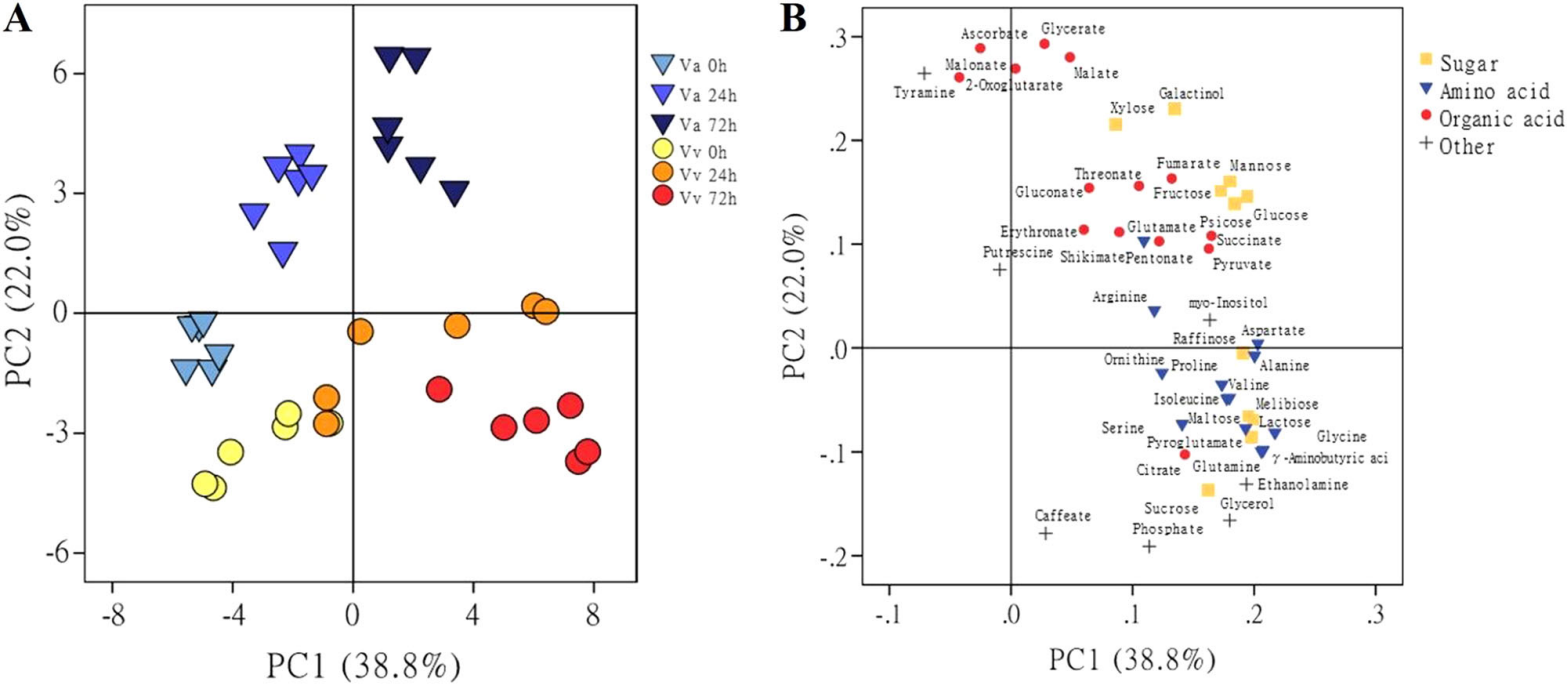
Principal component analysis of metabolite profiles in leaves of *V. amurensis* and *V. vinifera* cv. Muscat of Hamburg. **Va** *V. amurensis*; **Vv** *V. vinifera* cv. Muscat of Hamburg. Score plot (**a**) of samples and loading plot (**b**) of metabolites for the first two components, 1 (PC1) and 2 (PC2). Samples at 0, 24, and 72 h after cold treatment are represented by different shapes with colors. Each point represents an individual biological replicate in **a** (*n* = 6) and a single metabolite in **b**

No morphological difference was observed between *V. amurensis* and *V. vinifera* cv. Muscat of Hamburg under cold treatment (Supplementary Figure S2); nevertheless, the levels of metabolites changed in the two varieties after the cold acclamation. GC–TOF–MS was used to investigate the metabolic shifts resulting from cold stress in the two grape species. A total of 11 sugars, 13 amino acids, 14 organic acids, and 7 other compounds were detected. PCA was performed to test the metabolic changes in the two types of grapes under the cold condition (Fig. 1).

The PCA score plot indicated that the first principal component (PC1) and the second principal component (PC2) represented 38.8% and 22.0% of the total variance, respectively (Fig. 1a, Supplementary Table S2). The two varieties did not separate under the normal condition without stress in the PCA score plot. After 24 h of cold stress, both types of grapes clustered to the right of the pre-stress samples along PC1. However, their values differed along PC2, i.e., positive for *V. amurensis* and negative for Muscat of Hamburg. After 72 h of CA, the distinction between *V. amurensis* and Muscat of Hamburg remained consistent over the cold stress, moving farther along PC1 with additional cold stress and maintaining their clear separation in PC2.

The loading plot was performed to show the contribution of individual metabolites to the total variance (Fig. 1b, Supplementary Table S3). The metabolites with huge values along the first two components were considered as important variance of the loading plot. Sugars were identified as important in both dimensions (e.g., galactinol and fructose). Compounds that contributed to distinction primarily along PC1 were amino acids (e.g., aspartate and proline). Separation on PC2 could be attributed to organic acids (e.g., ascorbate and 2-oxoglutarate). Detailed comparisons of each metabolite inter- and intra-species were performed.

### Comparison of metabolite levels between *V. amurensis* and *V. vinifera* under the pre-stress condition

**Fig. 2:**
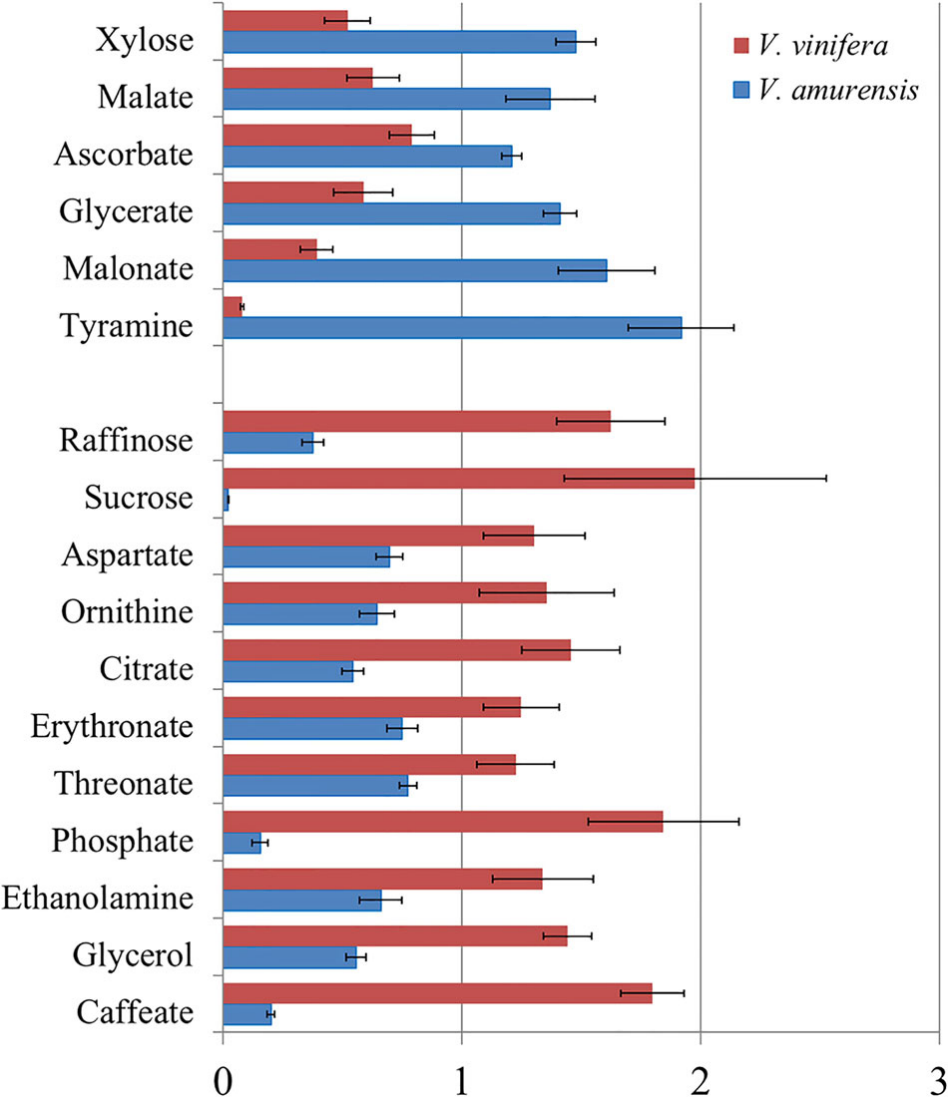
Metabolites with significantly different levels (*P*-value < 0.05, Student’s *t*-test) in *V. amurensis* (blue) and *V. vinifera cv*. Muscat of Hamburg (red) under the non-cold stress condition. Metabolite levels are normalized by the means of all samples and presented as the mean ± SEM. of six biological replicates

The relative contents of metabolites with significant differences (*P* < 0.05) between the two types of grapes under the normal condition are shown in Fig. 2. Compared with *V. vinifera*, *V. amurensis* had higher concentrations of six metabolites, namely, one carbohydrate (xylose), four organic acids (malate, ascorbate, glycerate, and malonate), and tyramine. By contrast, compared with *V. amurensis*, *V. vinifera* accumulated higher contents of 11 metabolites, namely, two carbohydrates (raffinose and sucrose), two amino acids (asparate and ornithine), three organic acids (citrate, erythronate, and threonate), and four other compounds (phosphate, ethanolamine, glycerol, and caffeate). Maltose was not detected under pre-stress conditions in either type of grape. No significant differences were found in the other identified metabolites between the two species (Supplementary Table S1).

### Common responding metabolites in *V. amurensis* and *V. vinifera* under cold treatment

**Fig. 3:**
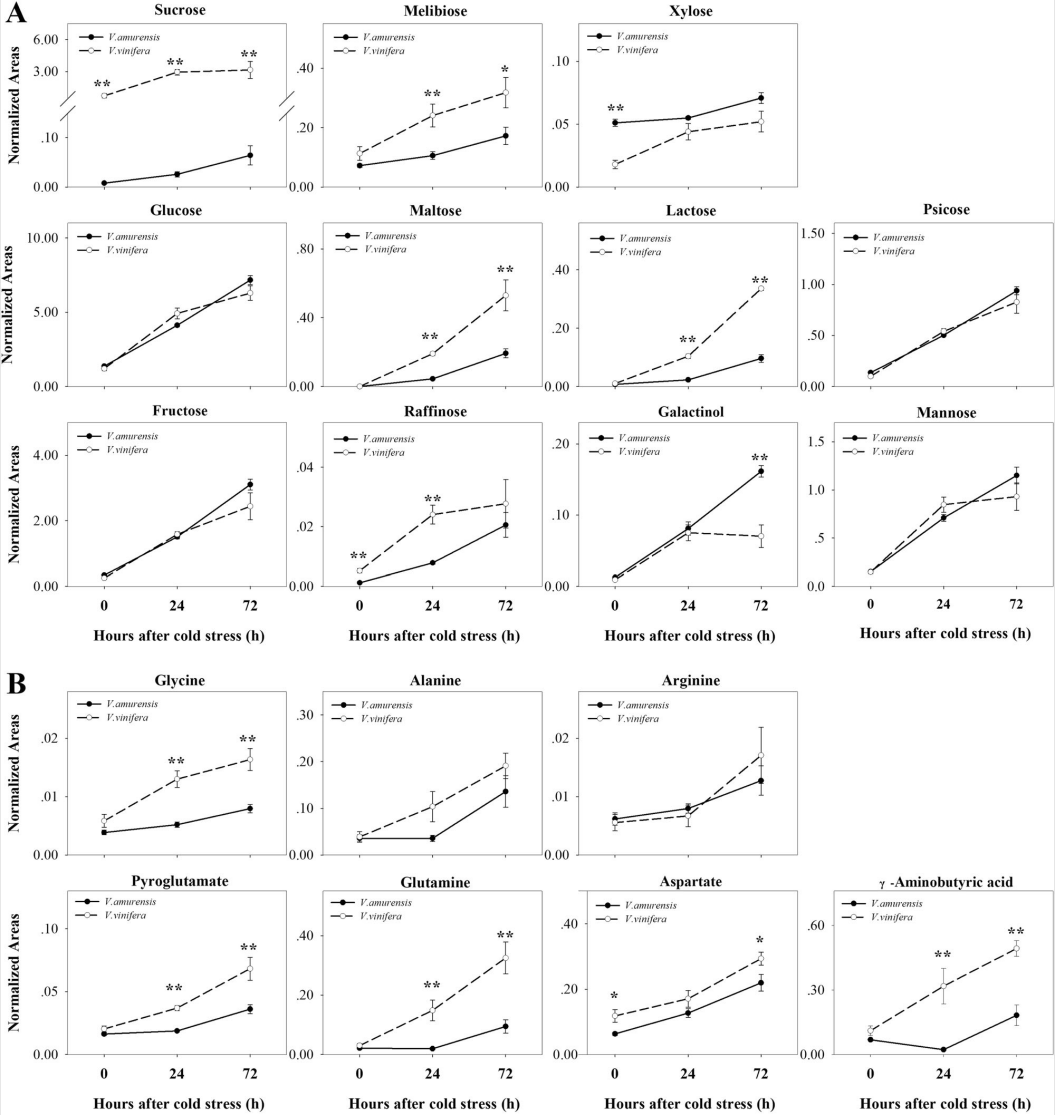

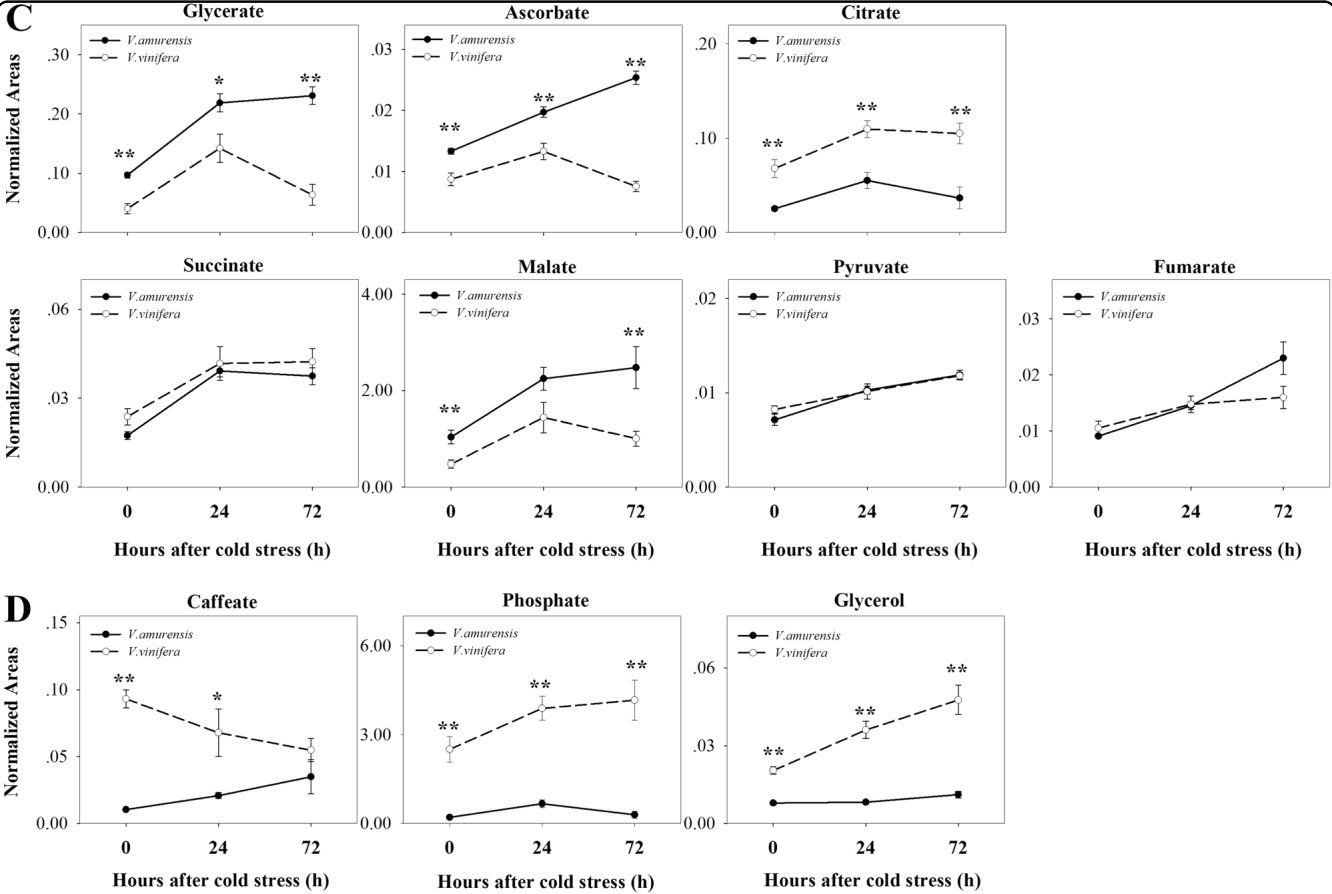
Common responding metabolites in *V. amurensis* and *V. vinifera* cv. Muscat Hamburg under the cold stress condition. **a** Sugars; **b** amino acids; **c** organic acids; **d** others. The time points are the non-stress condition (0) and cold stress at 24 h (24) and 72 h (72). The time points are connected using solid (*V. amurensis*) or dotted (*V. vinifera*) lines. Each data point represents the average of six biological replicates with error bars representing the standard deviation. Asterisks (**) and (*) indicate significant differences between the two species at *P*-value < 0.01 and *P*-value < 0.05 (Student’s t-test), respectively.

Eleven sugars increased significantly in both species after cold stress compared with levels in the normal condition (Fig. 3a). Sucrose and melibiose were sustained at higher levels under CA than those in pre-stress samples in *V. amurensis* and Muscat of Hamburg. The levels of glucose, maltose, lactose, and psicose increased with time in the two species of grapes. Furthermore, fructose, raffinose, galactinol, and mannose continuously accumulated with time in *V. amurensis* but were sustained in Muscat of Hamburg. In particular, the galactinol level increased in *V. amurensis* more than that in Muscat of Hamburg after exposure to cold stress.

Seven amino acids changed significantly after cold stress in both grapes (Fig. 3b). Six of these amino acids, namely, glycine, alanine, arginine, pyroglutamate, glutamine, and aspartate, showed identical variations, that is, they accumulated with time. Pyroglutamate and glutamine were significantly upregulated in Muscat of Hamburg at 24 h of cold stress, and these amino acids increased until 72 h of CA in *V. amurensis*. Aspartate increased with time in *V. amurensis* and was upregulated remarkably until 72 h of CA in Muscat of Hamburg. However, the levels of γ-aminobutyric acid (GABA) showed opposite trends in the two types of grape at 24 h of CA; GABA decreased in *V. amurensis* but increased in Muscat of Hamburg.

Seven organic acids showed increasing trends in both types of grapes under cold treatment (Fig. 3c). Glycerate level increased at 24 h under CA and sustained a high level in *V. amurensis*. Additionally, this level increased at 24 h but subsequently decreased to the no-stress level at 72 h in Muscat of Hamburg. Ascorbate accumulated with time in *V. amurensis* but was downregulated after 24 h in Muscat of Hamburg. Citrate showed a sudden accumulation at 24 h of cold stress in *V. amurensis* but sustained a high level in Muscat of Hamburg. Succinate and malate were sustained at a higher level under CA than that in pre-stress samples in both species. Pyruvate and fumarate accumulated with time in *V. amurensis*, whereas these acids accumulated until 72 h of CA in Muscat of Hamburg.

Additionally, phosphate, glycerol, and caffeate, which were categorized as other compounds, responded to cold in both types of grape (Fig. 3d). Caffeate increased in *V. amurensis* but decreased in Muscat of Hamburg under cold stress. Phosphate remarkably increased in both species at 24 h of cold stress. Glycerol was significantly upregulated in cold stress samples of Muscat of Hamburg and accumulated notably until 72 h of CA in *V. amurensis*.

### Metabolites specifically accumulated in *V. amurensis* or *V. vinife*ra under cold stress

**Fig. 4:**
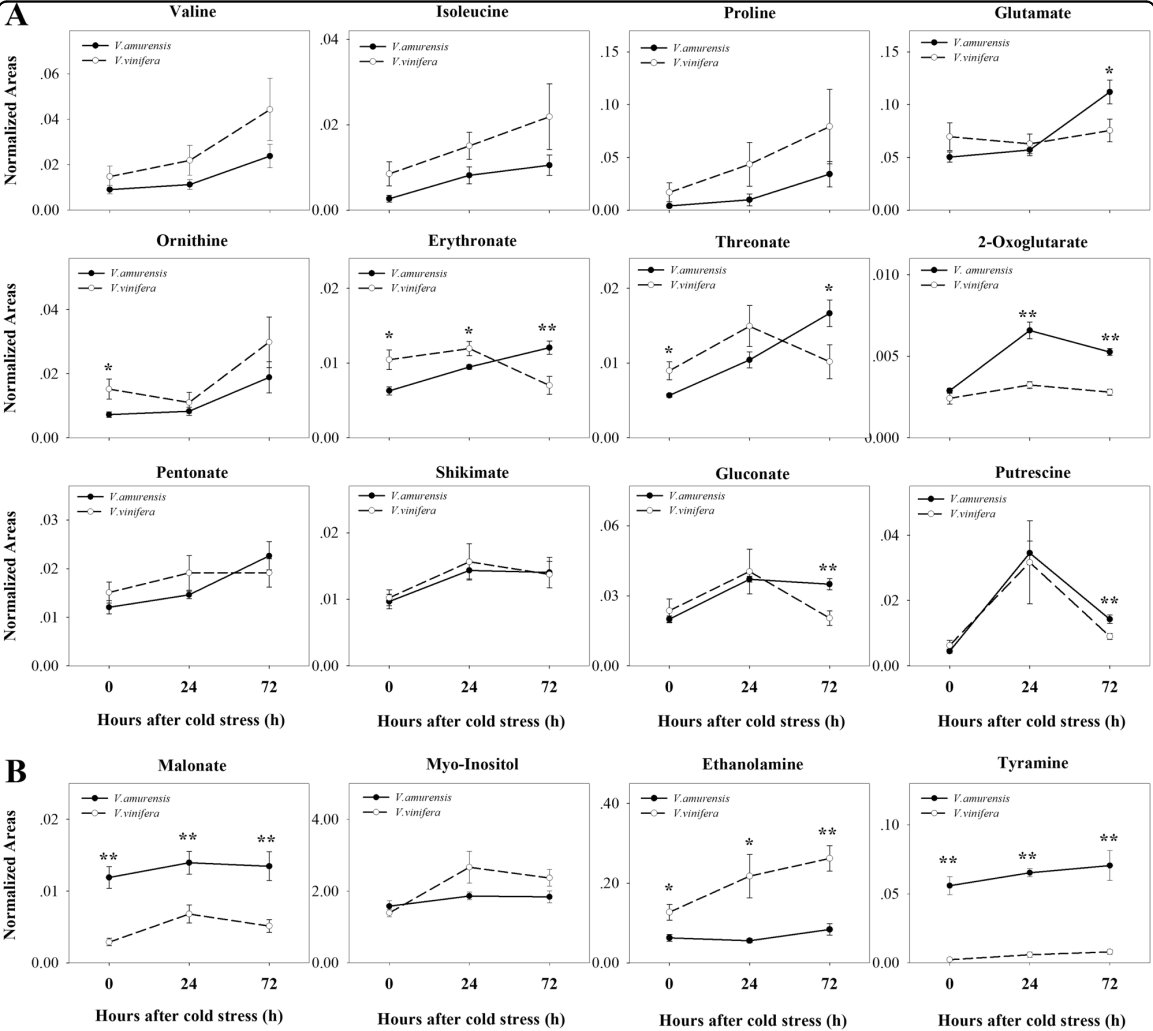
Metabolites that specifically accumulated in *V. amurensis* and *V. vinifera* cv. Muscat Hamburg under the cold stress condition. **a** the metabolites that specifically accumulated in* V. amurensis*; **b** the metabolites that specifically accumulated in *V. vinifera* cv. Muscat Hamburg. Time points are the non-stress condition (0) and cold stress at 24 h (24) and 72 h (72). Time points are connected using solid (*V. amurensis*) or dotted (*V. vinifera*) lines. Each data point represents the average of six biological replicates with error bars representing the standard deviation. Asterisks (**) and (*) indicate significant differences between the two species at *P*-value < 0.01 and *P*-value < 0.05 (Student’s t-test), respectively

Twelve metabolites were upregulated remarkably and specifically in *V. amurensis* under CA (Fig. 4a). These metabolites included five amino acids (isoleucine, valine, proline, ornithine, and glutamine), six organic acids (2-oxoglutarate, gluconate, erythronate, threonate, shikimate, and pentonate), and putrescine. Isoleucine began to increase at 24 h, whereas the other amino acids accumulated significantly at 72 h of CA. Proline increased by eightfold at 72 h of CA with the largest fold change of the amino acids. Organic acids increased significantly in *V. amurensis* from 24 h of cold treatment except for pentonate, which increased at 72 h of CA. Putrescine was induced up to eightfold at 24 h of CA but then decreased to threefold at 72 h in *V. amurensis*. The levels of putrescine and 2-oxoglutarate were higher in *V. amurensis* than those in Muscat of Hamburg under cold stress. No significant difference was found in levels of putrescine and 2-oxoglutarate between the two species under the no-stress condition.

Four metabolites increased significantly only in Muscat of Hamburg during CA (Fig. 4b). A carbohydrate derivative (myo-Inositol) increased with time under CA. Malonate was induced by cold at 24 h and then decreased at 72 h after CA. Malonate content was higher in *V. amurensis* than that in *V. vinifera* under pre-stress and cold treatment conditions. Ethanolamine was higher in *V. vinifera* than in *V. amurensis* under non-cold conditions, and tyramine was lower, with both induced at 72 h of CA in *V. vinifera*.

### Expression of genes encoding for BAMY, GolS, and RafS

**Fig. 5:**
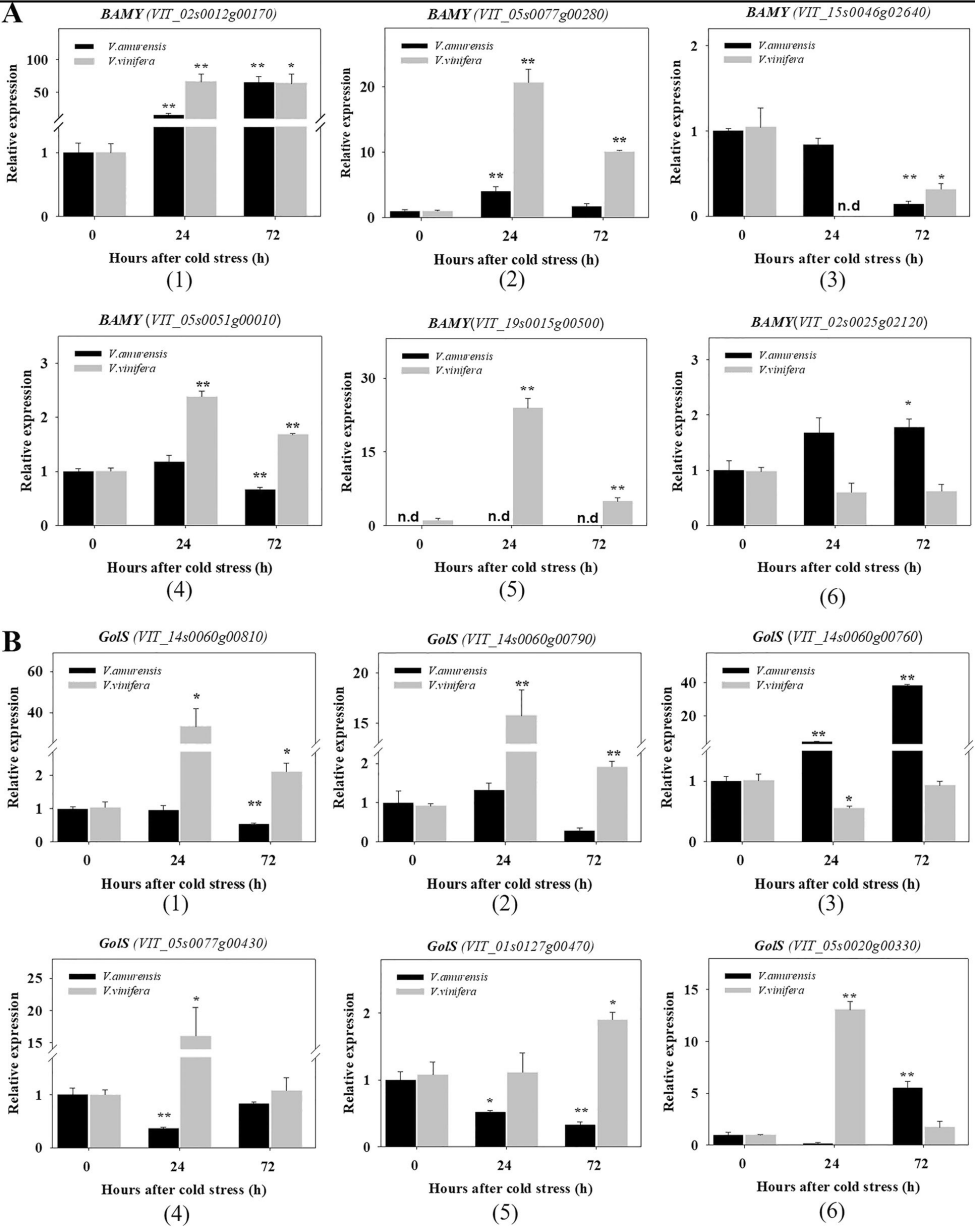

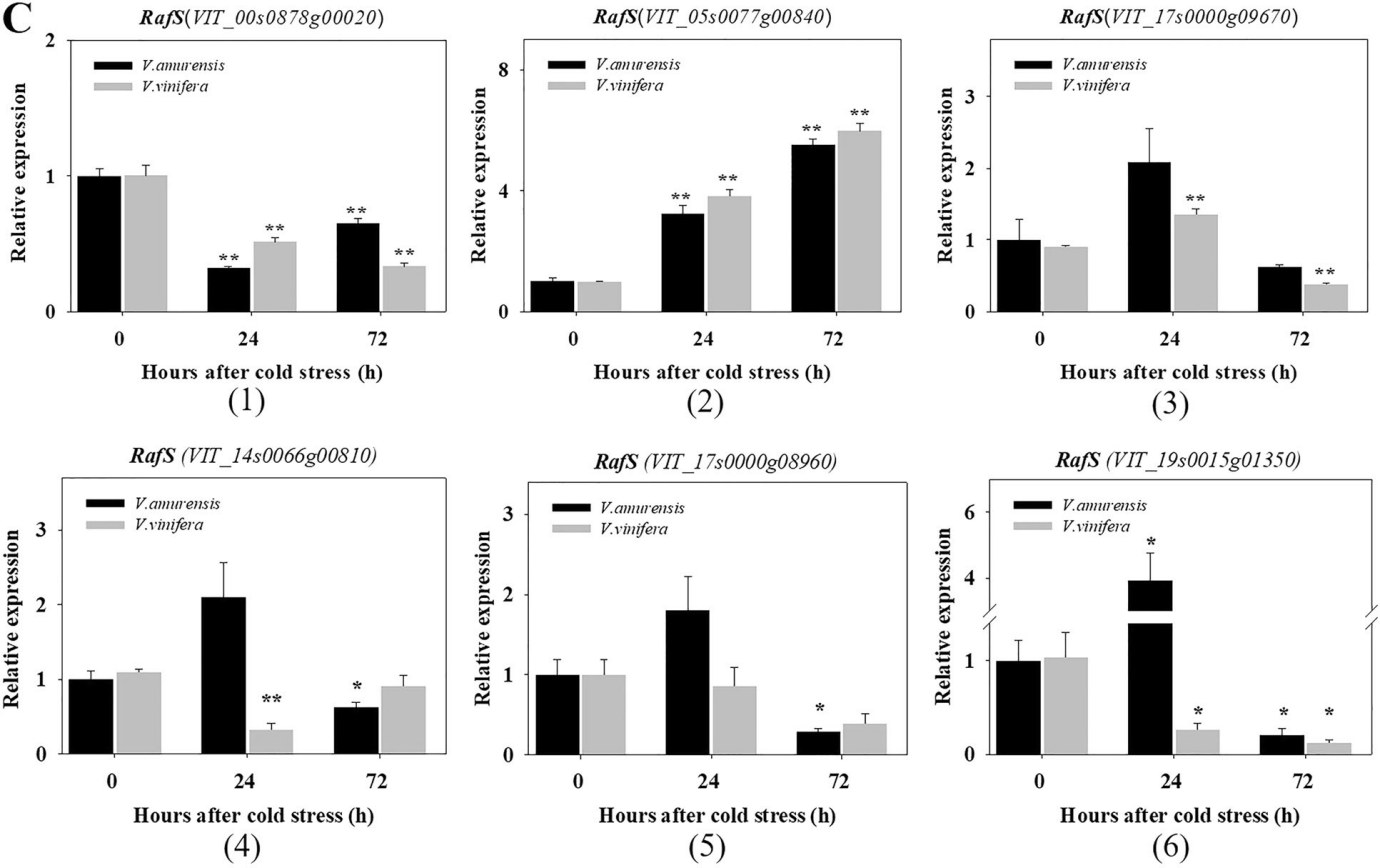
qRT-PCR results for the BAMY, GolS, and RafS gene family members in *V. amurensis*and *V. vinifera* cv. Muscat Hamburg. **a** BAMY; **b** GolS; **c** RafS. The time points are the non-stress condition (0) and cold stress at 24 h (24) and 72 h (72). Gene expression was normalized to the expression obtained in* V. amurensis* under the non-stress condition. The mean expression value was calculated from three technical replicates with three independent biological replicates (n = 3). Error bars indicate the standard error of the mean. Asterisks (**) and (*) indicate significant differences compared with the WT at *P*-value < 0.01 and *P*-value < 0.05 (Student’s t-test), respectively

Raffinose and galactinol significantly increased in the two species of grapes under cold stress. Raffinose family oligosaccharides (RFOs) are associated with chilling-stress tolerance in *Arabidopsis* and serve as plant cell protectants from oxidative damage^48,49^. GolS and RafS are key enzymes in the synthesis of galactinol and raffinose, respectively. Similarly, maltose accumulates in *Arabidopsis* under cold, and BAMY is the key enzyme in the synthesis of maltose^50^. Maltose was not detected in *V. amurensis* and Muscat of Hamburg under the normal condition but accumulated clearly after cold stress. Genes encoding for BAMY, GolS, and RafS were chosen to discover the relationships between metabolite accumulation and gene expression. The expression patterns of each gene family were analyzed by qRT-PCR, which were based on the same time points as those of the metabolic analysis (Fig. 5). The genes that were very low or undetectable in samples are not shown.

More members of BAMY were induced by cold in *V. vinifera* than in *V. amurensis* (Fig. 5a). *VIT_02s0012g00170* displayed an upregulated and sustained high expression under cold stress (1). *VIT_05s0077g00280* showed a sudden increase at 24 h in *V. amurensis*, whereas it was induced at both time points of cold stress in Muscat of Hamburg (2). The expression of *VIT_05s0051g00010* and *VIT_19s0015g00500* was significantly elevated in Muscat of Hamburg by cold (4 and 5). *VIT_15s0046g02640* expression exhibited no change at 24 h and then prominently decreased at 72 h in both species (3). *VIT_02s0025g02120* induced uniquely at 72 h in *V. amurensis* by cold stress (6).

The expression patterns of GolS are exhibited in Fig. 5b. The genes *VIT_14s0060g00810*, *VIT_14s0060g00790*, *VIT_05s0077g00430*, and *VIT_05s0020g00330* showed the same expression patterns in Muscat of Hamburg, with expression levels increased significantly at 24 h and subsequently decreased at 72 h (1, 2, 4, 6). *VIT_14s0060g00760* was significantly and sustainably increased in *V. amurensis* under the cold condition (3). The transcription level of *VIT_01s0127g00470* increased at 72 h in Muscat of Hamburg, whereas the level decreased in *V. amurensis* (5).

Members subjected to qRT-PCR in the RafS gene family are shown in Fig. 5c. *VIT_00s0878g00020*, *VIT_14s0066g00810*, and *VIT_17s0000g08960* were not significantly different or were downregulated in *V. amurensis* and Muscat of Hamburg in response to cold (1, 4, 5). The levels of *VIT_05s0077g00840* increased significantly and were sustained by cold in both species (2)*. VIT_17s0000g09670* displayed a sudden increase at 24 h and then decreased in both grapes (3). *VIT_19s0015g01350* remarkably increased at 24 h in *V. amurensis* and subsequently decreased at 72 h of cold treatment, whereas it decreased in Muscat of Hamburg during cold treatment (6).

## Discussion

In this study, we used GC–TOF–MS to compare the metabolic profiles of *V. amurensis* and Muscat of Hamburg under control and cold stress conditions. This analysis highlighted the specific cold-responding metabolites in *V. amurensis*. The results of the analysis were used to investigate the mechanisms underlying low temperature adaptation in grapes.

The compounds accumulated in both grapes species during CA represented fundamental metabolites responding to cold stress. These metabolites, which included sugars (e.g., sucrose and glucose), amino acids (e.g., glycine and aspartate), and organic acids (succinate and malate), contributed to cold stress response/tolerance. Some of these metabolites are known to accumulate in other plants^13,29^. Cold exposure of *Arabidopsis* increases the levels of amino acids (glycine and arginine), the carbohydrates glucose and maltose, TCA cycle intermediates, and succinate^29^. Glucose rapidly accumulates in both cold-tolerant and -intolerant accessions of *Arabidopsis* and remains high during 7 days of CA^51^. Metabolites glycine, aspartate, glucose, and sucrose also accumulate in rice after CA^21^. Changes in these metabolites suggest conserved reconstruction in metabolome levels in plants to increase cold tolerance.

Different patterns of accumulation were also found in a subset of common responding metabolites between the two species. For example, galactinol, raffinose, fructose, mannose, glycine, and ascorbate were induced by cold stress in *V. amurensis* but remained constant after 24 h of cold exposure in Muscat of Hamburg. In wheat leaves, mannose modulates the expression of the enzymatic antioxidant defense system^52^, and d-mannose treatment influences the expression level of different antioxidant enzymes, such as ascorbate peroxidase (APX), peroxidase (POD), and catalase (CAT) in wheat coleoptiles^53^. Glycine is directly related to the metabolism of amino acids, and upregulation of glycine levels could result from the recycling of amino acids^54^. In particular, amounts of galactinol and ascorbate were higher in *V. amurensis* than those in Muscat of Hamburg. Raffinose and galactinol are precursors of RFOs that most likely act as ROS scavengers and osmoprotectants to stabilize cellular membranes against abiotic stresses, including chilling^48^. Raffinose is induced at low temperatures in dicots^51^ and monocots^55^. High levels of galactinol during CA correlate with low temperature tolerance in diploid strawberry^24^. Ascorbate as an abundant antioxidant can protect against reactive oxygen species, which are produced when plants are exposed to low temperature stress^56^. Metabolites that were sustainably upregulated and in high amounts in *V. amurensis* may protect plants from long-term low temperature damage and increase cold tolerance.

A subset of metabolites specifically accumulated in *V. amurensis* (Fig. 4a). Of these specific compounds, the level of putrescine increases in several plants responding to multiple abiotic stresses, such as salt^57^, drought^58^, and low temperature^59^. Exogenous application of putrescine decreases membrane leakage that is caused by low temperature in tomato^60^. Mutants of *Arabidopsis* (*ADC2*) that are deficient in endogenous putrescine are sensitive to abiotic stresses^61^. In our research, high levels and specific accumulation of putrescine might relate to the cold tolerance of *V. amurensis*. For several amino acids, including valine, isoleucine, and proline, the accumulation was greater in *V. amurensis* than that in Muscat of Hamburg during cold stress. Free amino acids are hypothesized to play a role as osmolytes during abiotic stress responses in plants^62^. In particular, the accumulation of branched-chain amino acids, such as valine and isoleucine, reportedly show a high fold increase under abiotic stress^63^. Proline, as an osmoprotectant, accumulates during CA^26,46^. In previous research examining exogenous proline treatment, a positive correlation was revealed between proline accumulation and cold stress tolerance^64^. The level of proline is higher in the wheat crown than that in shoots, which is an indication of an essential role in the frost tolerance of the crown^23^. The specific accumulation of these metabolites in cold-acclimated *V. amurensis* provided evidence for their important roles in defense against cold stress. In particular, the levels of 2-oxoglutarate and putrescine were higher in *V. amurensis* than those in Muscat of Hamburg. This result indicated that these metabolites act as indicator compounds of the cold tolerance of grape. Moreover, the accumulation of different metabolites demonstrated that different response mechanisms are required in various stages of cold stress in *V. amurensis*.

Expression analysis of the genes associated with the accumulated metabolites was performed. A relationship between high transcript levels of the gene counterparts and increased levels of metabolites has been reported in cold-treated *Arabidopsis*^65^ and rice^21^. In the present study, *VIT_02s0012g00170* of the BAMY gene family displayed an upregulated and sustained high expression under cold stress (Fig. 5a1). The upregulated expression pattern of this gene was consistent with the accumulation of maltose under cold stress. Therefore, this gene was likely the predominant gene for maltose accumulation in grapes under the cold condition. Moreover, the higher content of maltose in Muscat of Hamburg than that in *V. amurensis* suggested that more members of BAMY were induced by cold in Muscat of Hamburg. We observed that different members of GolS were upregulated in the two species under cold. *VIT_14s0060g00760* showed a significant and sustained increase in *V. amurensis* (Fig. 5b3), which was consistent with the high accumulation of galactinol in *V. amurensis*, reflecting its potential role in galactinol biosynthesis in *V. amurensis* under the cold condition. By contrast, in Muscat of Hamburg, the accumulation of galactinol might be the result of the upregulated expression of several GolS genes during cold. For RafS, the expression of *VIT_05s0077g00840* was significantly promoted during cold stress, and this trend was consistent with the biosynthesis of raffinose in grape (Fig. 5c1). Therefore, this gene might be the primary contributor to the accumulation of raffinose in grape under cold stress. *VIT_19s0015g01350* was significantly upregulated in cold-treated *V. amurensis* (Fig. 5c6) and therefore was also a contributing gene to the accumulation of raffinose in *V. amurensis* under CA. These results provide new insights into the relationship between transcript level and metabolite accumulation and into the mechanisms underlying metabolite accumulation under cold exposure.

## Electronic supplementary material

read me

Figure S1

Figure S2

Table S1

Table S2

Table S3

Table S4

Table S5
