# Supplementary material for: Comparative metabolic profiling of Vitis amurensis and Vitis vinifera during cold acclimation
Source: Hortic Res. 2019 Jan 1;6:8. doi: 10.1038/s41438-018-0083-5 (PMC6312538; doi:10.1038/s41438-018-0083-5)
Supplement: Supplementary file 1 — read me [file 41438_2018_83_MOESM1_ESM.docx]

**SUPPLEMENTARY DATA**

**Table S1.** Profiling results of all identified metabolites.

**Table S2.** PCA component score of samples were detected.

**Table S3.** PCA component loadings of total metabolites were detected.

**Table S4.** List of GolS and RafS gene family members.

**Table S5.** List of primer sequences used for qRT-PCR.

**Figure S1.** Phylogenetic analysis of BAMY gene family members in *V. vinifera* and *Arabidopsis*.

**Figure S2.** The morphological phenotype of *V. amurensis* and *V. vinifera* cv. *Muscat of Hamburg* with non-cold stress (0 h) and under 24 h and 72 h cold stress.

.
