# Supplementary material for: Comparative metabolic profiling of Vitis amurensis and Vitis vinifera during cold acclimation
Source: Hortic Res. 2019 Jan 1;6:8. doi: 10.1038/s41438-018-0083-5 (PMC6312538; doi:10.1038/s41438-018-0083-5)

**Figure S1.** **Phylogenetic analysis of BAMY gene family members in *V. vinifera* and *Arabidopsis*.**

Phylogenetic tree was constructed by MEGA7 with Neighbour-Joining method and bootstrap of 1000 replications. Values less than 40 were cut off. The BAMY protein sequences of *A. thaliana* used in this study were obtained from NCBI (http://www.ncbi.nlm.nih.gov/).


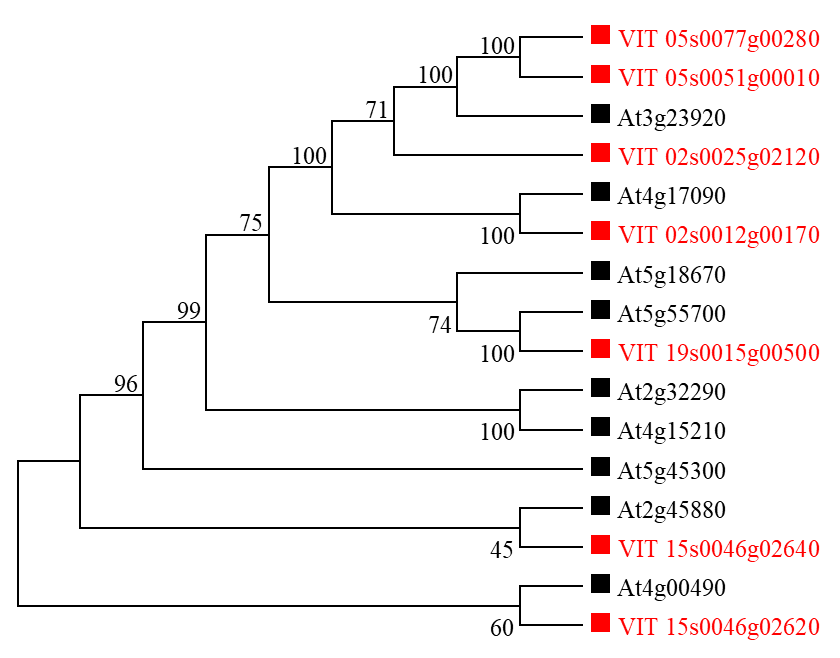

Supplement: Supplementary file 2 — Figure S1 [file 41438_2018_83_MOESM2_ESM.docx]
