# Supplementary material for: Comparative metabolic profiling of Vitis amurensis and Vitis vinifera during cold acclimation
Source: Hortic Res. 2019 Jan 1;6:8. doi: 10.1038/s41438-018-0083-5 (PMC6312538; doi:10.1038/s41438-018-0083-5)

**Figure S2.** **The morphological phenotype of *V. amurensis* and *V. vinifera* cv. *Muscat of Hamburg* with non-cold stress (0 h) and under 24 h and 72 h cold stress.**

There was no morphological difference between *V. amurensis* and *V. vinifera* cv. Muscat of Hamburg after the cold treatment.


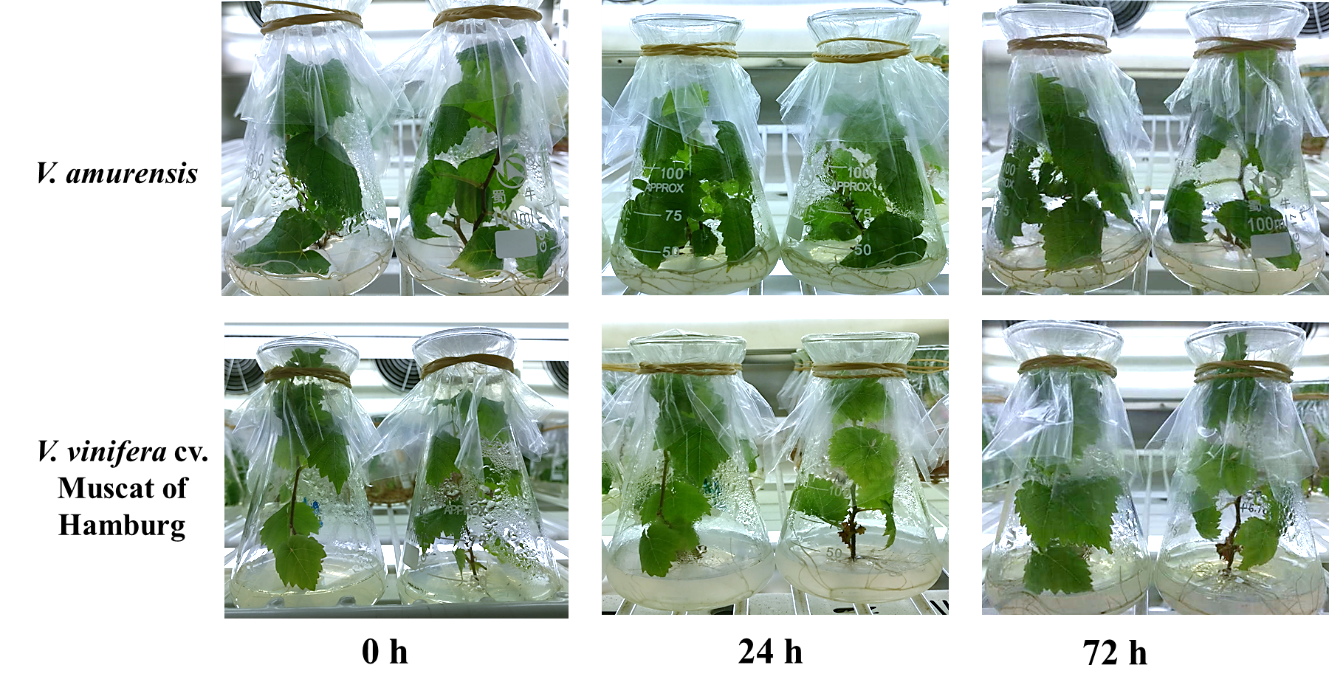

Supplement: Supplementary file 3 — Figure S2 [file 41438_2018_83_MOESM3_ESM.docx]
