# Supplementary material for: Comparative metabolic profiling of Vitis amurensis and Vitis vinifera during cold acclimation
Source: Hortic Res. 2019 Jan 1;6:8. doi: 10.1038/s41438-018-0083-5 (PMC6312538; doi:10.1038/s41438-018-0083-5)
Supplement: Supplementary file 4 [file 41438_2018_83_MOESM4_ESM.docx]

Table S1. Profiling results of all identified metabolites. Values are the means ± SEM of six biological replicates.

			Vitis amurensis									*Vitis vinifera* cv. Muscat of Hamburg									T test of metabolites levels between two grapes at the same time point		
			0h		24h		72h		P value			0h		24h		72h		P value			P value		
NO.	Metabolite Class	Coresponding Metabolite	Means	SEM	Means	SEM	Means	SEM	24/0	72/0	72/24	Means	SEM	Means	SEM	Means	SEM	24/0	72/0	72/24	0 h	24 h	72 h
1	Sugars	Sucrose	0.008088	0.000828	0.025989	0.007047	0.063966	0.006798	0.007456218**	0.015719440704297*	0.085934966	0.720412	0.006606	2.944372	0.006427	3.464526	0.005714	0.0000431**	0.0148891772092547*	0.809200769	0.00522423572644457	4.35960599927959E-07*	0.003260058**
2	Sugars	Fructose	0.337553	0.025650	1.504793	0.204955	3.105032	0.232352	0.0000000554**	0.000000014**	0.00000377**	0.248849	0.246533	1.581631	0.260878	2.438242	0.204175	0.0000000616**	0.000353904900320444**	0.068867544	0.10675889	0.470935222	0.164021822
3	Sugars	Glucose	1.366575	0.105247	4.116783	0.417632	7.166525	0.542997	0.0000000056**	0.00000000463**	0.00000215**	1.198585	0.595092	4.914394	0.571043	6.292681	0.439203	0.00000216**	1.58359744471499E-06**	0.0462159340353435*	0.362260586	0.058660554	0.157348567
4	Sugars	Raffinose	0.001200	0.000142	0.007848	0.001375	0.020573	0.001595	0.000000207**	0.000842802**	0.011952104*	0.005167	0.001573	0.024026	0.001491	0.027691	0.001221	0.000162331**	0.0202805731342936*	0.683464556	0.00029713016440353	0.000479556198523335*	0.453475957
5	Sugars	Galactinol	0.012406	0.002002	0.080936	0.016406	0.161391	0.016908	0.0000287**	0.00000000733**	0.0000696**	0.009079	0.017142	0.075060	0.017792	0.070202	0.014298	0.000164898**	0.00338643857889297**	0.807950128	0.229216354	0.694517387	0.000470972**
6	Sugars	Melibiose	0.072580	0.006518	0.106074	0.010001	0.172276	0.010366	0.042859525*	0.007907675**	0.06630661	0.113223	0.008617	0.241156	0.013701	0.317925	0.012609	0.016782669*	0.00426732100880505**	0.255612271	0.117197138	0.00753618181372017**	0.0324721758*
7	Sugars	Maltose	NA	NA	0.044623	NA	0.192696	NA	0.00000903**	0.0000202**	0.000211335**	NA	NA	0.190727	NA	0.529497	NA	0.00000000846**	0.000153788063574181**	0.00385576781312217	NA	3.06911941380656E-07*	0.004838951**
8	Sugars	Mannose	0.151257	0.008540	0.709723	0.095711	1.149939	0.110537	0.0000000195**	0.000000444**	0.000802373**	0.145958	0.116208	0.846460	0.125268	0.930177	0.095717	0.00000546**	0.000297556928561152**	0.620848315	0.783894633	0.141069497	0.220228074
9	Sugars	Lactose	0.007521	0.001099	0.022993	0.003378	0.096294	0.003979	0.001117531**	0.0000579**	0.000326525**	0.010378	0.003731	0.103732	0.004669	0.335622	0.003976	0.000000198**	8.34749595796272E-14**	2.52998733455937E-1	0.106353059	1.55361755447783E-06*	0.0000000139**
10	Sugars	Xylose	0.051108	0.002868	0.054962	0.002941	0.070848	0.002232	0.247793226	0.003746208**	0.00607472**	0.018055	0.002234	0.044057	0.001629	0.052075	0.001799	0.005646507**	0.00319699346528595**	0.463590837	0.00002022289932098	0.136863298	0.07081415
11	Sugars	Psicose	0.135740	0.008378	0.501440	0.061263	0.937737	0.071653	0.0000000000657**	0.00000000232**	0.000000842**	0.099324	0.077343	0.539268	0.076730	0.826162	0.059615	0.0000000469**	0.0000594346111418192**	0.0282016588017717*	0.060511989	0.206151129	0.35785188
12	Amino acids	Alanine	0.035849	0.007422	0.035778	0.007613	0.136086	0.008207	0.994507849	0.016301663*	0.015917135*	0.038973	0.007570	0.103777	0.006796	0.191152	0.007375	0.089472653	0.000396882543968657**	0.065322466	0.822853821	0.067820664	0.232613845
13	Amino acids	Valine	0.009088	0.001881	0.011324	0.001795	0.023847	0.001940	0.454854724	0.021988993*	0.047892466*	0.014842	0.001798	0.021973	0.001250	0.044344	0.001403	0.397601215	0.068985297	0.172270409	0.278315496	0.15602935	0.191673452
14	Amino acids	Isoleucine	0.002698	0.000764	0.008170	0.000971	0.010544	0.001762	0.027909586*	0.01040748*	0.460925204	0.008517	0.001629	0.015069	0.001343	0.021900	0.001358	0.150974039	0.131948531	0.428920749	0.072583206	0.093717128	0.187319076
15	Amino acids	Proline	0.004065	0.001720	0.009905	0.001708	0.034158	0.003272	0.344684468	0.032905008*	0.098120155	0.016952	0.003236	0.043600	0.003160	0.079260	0.003268	0.26539696	0.117701649	0.40368607	0.190400998	0.1475252	0.253976818
16	Amino acids	Glycine	0.003858	0.000395	0.005210	0.000691	0.007952	0.000611	0.043970944*	0.000565545**	0.008719777**	0.005849	0.000538	0.013013	0.000494	0.016357	0.000436	0.002850625**	0.000726983991448804**	0.188907832	0.124448129	0.000412778063011716*	0.001927688**
17	Amino acids	Serine	0.018521	0.005391	0.012142	0.005809	0.023882	0.005339	0.349331379	0.454985183	0.063472232	0.037210	0.005128	0.043220	0.005079	0.057662	0.004835	0.817539466	0.48171796	0.616053672	0.343547293	0.119262555	0.153233417
18	Amino acids	Aspartate	0.063191	0.005111	0.126609	0.011186	0.219107	0.017583	0.001139129**	0.000144617**	0.009534326**	0.117878	0.015833	0.170369	0.012956	0.293290	0.011273	0.13166697	0.0000901951446910219**	0.00360462771919926	0.0206868132655208*	0.158251013	0.046840271*
19	Amino acids	Pyroglutamate	0.016277	0.001218	0.018743	0.001314	0.036033	0.000950	0.154844908	0.000356868**	0.000854979**	0.020284	0.000913	0.036842	0.001188	0.068162	0.001109	0.000269848**	0.00047454216161196**	0.00733509206239833	0.153819606	0.0000102673922064842	0.008280629**
20	Amino acids	γ-Aminobutyric acid (G	0.068221	0.003402	0.022414	0.010958	0.182145	0.011748	0.00000649**	0.039391809*	0.007791699**	0.110538	0.012658	0.317665	0.011971	0.493133	0.008182	0.035186469*	4.29865052238717E-06**	0.079251991	0.09032009	0.00491738225829258**	0.000422845**
22	Amino acids	Glutamine	0.021410	0.003779	0.019512	0.002769	0.094173	0.003084	0.692437499	0.010135315*	0.008502963**	0.029842	0.002922	0.148259	0.002857	0.324926	0.002732	0.007515429**	0.000278560334738115**	0.0200749899305527*	0.301439312	0.0042078608448181**	0.002685522**
23	Amino acids	Ornithine	0.007224	0.000817	0.008252	0.000842	0.018861	0.000720	0.508613497	0.039599112*	0.060355926	0.015186	0.000729	0.010973	0.000936	0.029861	0.001016	0.369205067	0.115314331	0.050725875	0.0352194856733049*	0.443376273	0.26275841
24	Amino acids	Arginine	0.006192	0.001045	0.007977	0.001134	0.012751	0.001128	0.206018111	0.036250657*	0.099730997	0.005575	0.000855	0.006703	0.000856	0.017081	0.000835	0.636442687	0.0440563326100777*	0.070713433	0.732687427	0.538816959	0.44195538
25	Amino acids	Pyruvate	0.007118	0.000612	0.010270	0.000645	0.011890	0.000631	0.001524587**	0.000146173**	0.033733329*	0.008222	0.000429	0.010131	0.000410	0.011796	0.000420	0.056274335	0.0000527806301759981**	0.086967583	0.157520822	0.879286029	0.886275775
26	Organic acids	Succinate	0.017353	0.001333	0.039195	0.004025	0.037417	0.004200	0.00000385**	0.0000795**	0.620445165	0.023726	0.004506	0.041724	0.004960	0.042316	0.004315	0.017580499*	0.00519085297097944**	0.936085417	0.066736163	0.683932177	0.372643421
27	Organic acids	Glycerate	0.096862	0.004745	0.218793	0.017979	0.230864	0.019782	0.0000165**	0.00000662**	0.582762873	0.040319	0.021249	0.142249	0.024287	0.063696	0.022197	0.002392845**	0.259162675	0.0243766824842477*	0.00015983680147650	0.0218795900965681*	0.0000283**
28	Organic acids	Fumarate	0.009073	0.000352	0.014494	0.001055	0.022991	0.001116	0.0000652**	0.000810473**	0.018335029*	0.010515	0.001140	0.014748	0.001028	0.015979	0.001133	0.052666424	0.0437218829166283*	0.634783296	0.28435256	0.881665494	0.077073305
29	Organic acids	Malonate	0.011904	0.001495	0.013954	0.001854	0.013468	0.001996	0.369810042	0.545614464	0.852918187	0.002905	0.001945	0.006822	0.001988	0.005145	0.001882	0.017315566*	0.057335709	0.30975535	0.00020018328219155	0.00573925953187I9**	0.00357102**
30	Organic acids	Malate	1.035517	0.140281	2.245079	0.355558	2.476117	0.326878	0.001334238**	0.010632938*	0.652372495	0.474840	0.330839	1.439890	0.358612	1.002554	0.316886	0.014815654*	0.0131518165746999*	0.243804786	0.00638113781637062	0.069373188	0.009920013**
31	Organic acids	Erythronate	0.006278	0.000548	0.009464	0.000690	0.012043	0.000588	0.000491653**	0.000262827**	0.021394313*	0.010441	0.000585	0.011922	0.000554	0.006999	0.000565	0.385030915	0.083071282	0.00946421929731539	0.0155402170389952*	0.0345358671905674*	0.007209437**
32	Organic acids	Threonate	0.005667	0.000264	0.010404	0.001186	0.016652	0.001151	0.001681207**	0.000111303**	0.013144398*	0.008961	0.001174	0.014938	0.001156	0.010176	0.001217	0.074600136	0.6451419	0.211169353	0.0228249572409204*	0.156495489	0.04790961*
33	Organic acids	2-Oxoglutarate	0.002885	0.000149	0.006581	0.000930	0.005254	0.000914	0.0000369**	0.00000369**	0.036308063*	0.002412	0.000940	0.003233	0.000924	0.002795	0.000778	0.073145954	0.359741692	0.146697931	0.245461491	0.000112909781036512*	0.00000557**
34	Organic acids	Pentonate	0.012057	0.001331	0.014631	0.000885	0.022671	0.001067	0.124997161	0.007650281**	0.023085218*	0.015102	0.001086	0.019135	0.001101	0.019173	0.000976	0.354885869	0.290888602	0.993653883	0.255910833	0.244499365	0.417621992
35	Organic acids	Shikimate	0.009672	0.001092	0.014351	0.001771	0.014041	0.001704	0.021227094*	0.11597467	0.909038985	0.010237	0.001579	0.015634	0.001550	0.013733	0.001437	0.099546373	0.163819373	0.584807977	0.735758	0.680148659	0.92118306
36	Organic acids	Citrate	0.025297	0.002103	0.055212	0.009549	0.036595	0.009311	0.007144264**	0.366515317	0.230618051	0.067982	0.009146	0.109648	0.010147	0.105053	0.009669	0.010096478*	0.0296195077435215*	0.754343379	0.0014321261418678*	0.001433662226 2783**	0.001711541**
37	Organic acids	Ascorbate	0.013325	0.000462	0.019723	0.001117	0.025382	0.001082	0.0000683**	0.00000148**	0.002390476**	0.008737	0.001270	0.013305	0.001361	0.007567	0.001256	0.023567383*	0.402779766	0.00521830017904215	0.00226957841398613	0.00267169960261033**	0.000000164**
38	Organic acids	Glutamate	0.050322	0.004761	0.057102	0.004633	0.111961	0.005108	0.371855191	0.000484107**	0.001321953**	0.069680	0.004112	0.062985	0.003024	0.075555	0.002976	0.688641722	0.73789105	0.396396984	0.200442986	0.597622655	0.040560938*
38	Organic acids	Gluconate	0.020030	0.001281	0.037126	0.003128	0.035021	0.003169	0.00000221**	0.00022818**	0.445693594	0.023595	0.003223	0.040438	0.003195	0.020462	0.002301	0.152856084	0.611083671	0.076608089	0.512135669	0.739905724	0.003948056**
39	Others	myo-Inositol	1.580266	0.151309	1.864755	0.140979	1.841915	0.119853	0.157224027	0.274415951	0.911363829	1.394025	0.089485	2.667050	0.140446	2.368724	0.117124	0.019241072*	0.00349254341259642**	0.566087958	0.335018102	0.110206634	0.097786232
40	Others	Putrescine	0.004449	0.000648	0.034570	0.005953	0.014255	0.007015	0.0000101**	0.0000486**	0.00037124**	0.006225	0.007385	0.031669	0.006862	0.008984	0.005547	0.07461651	0.140863566	0.104949299	0.304321759	0.830562325	0.006556653**
41	Others	Ethanolamine	0.062784	0.008339	0.055843	0.008175	0.083968	0.007821	0.498442057	0.229716088	0.09509172	0.127263	0.007488	0.217805	0.007730	0.262119	0.006060	0.149766842	0.00518325747803236**	0.499832164	0.0137736852644574*	0.0143320900166478*	0.000495063**
42	Others	Phosphate	0.211632	0.044929	0.668835	0.137197	0.299076	0.129457	0.007497231**	0.525315405	0.066892563	2.505984	0.122885	3.886242	0.148807	4.161968	0.146057	0.040514358*	0.06538263	0.73283233	0.00033822242876308	0.000017485471138754	0.000220895**
43	Others	Caffeate	0.010439	0.000847	0.020900	0.002465	0.034945	0.002878	0.001352966**	0.08234109	0.300366028	0.093140	0.002864	0.067884	0.002469	0.054838	0.001955	0.214989979	0.00595002229307813**	0.524909254	0.00000028569520914	0.0257311744592432*	0.223688537
44	Others	Glycerol	0.007903	0.000598	0.008230	0.000767	0.011191	0.000594	0.722463815	0.041950426*	0.066897749	0.020477	0.000720	0.036087	0.000670	0.047671	0.000662	0.00152754**	0.00096569050766409**	0.110652979	0.00001035377797127	9.31658689798717E-06*	0.0000988**
45	Others	Tyramine	0.055935	0.006435	0.065357	0.004813	0.070574	0.004842	0.212361715	0.269256891	0.649302864	0.002355	0.005438	0.005978	0.005635	0.008016	0.003781	0.099387932	0.00913313827138035**	0.457811877	8.32433699650245E-0	1.19564876037766E-08*	0.000184497**

*Statistical difference was determined by Student’s t-test t-test (**P < 0.01, *P < 0.05, n = 6) using R program.
